# Duodenoduodenostomy as an Attractive Option for Exocrine Drainage in Pancreas Transplantation: Insights From a Single-Center Cohort

**DOI:** 10.3389/ti.2025.15430

**Published:** 2025-11-03

**Authors:** Alba Torroella, Rongrong Hu Zhu, Carlos Castillo-Delgado, Marco Pavesi, Ramón Rull, Emma Folch-Puy, Rocío García, Clara Bassaganyas, Carles Pérez-Serrano, Pedro Ventura-Aguiar, Enrique Montagud-Marrahi, Víctor Emilio Holguin, Antonio J. Amor, Fritz Diekmann, Ángeles García-Criado, Juan Carlos García-Valdecasas, Josep Fuster, Joana Ferrer-Fàbrega

**Affiliations:** ^1^ Hepatobiliopancreatic Surgery and Liver and Pancreatic Transplantation Unit, Department of Surgery, Clinical Institute of Digestive and Metabolic Diseases (ICMDiM), Hospital Clínic Barcelona, Barcelona, Spain; ^2^ University of Barcelona, Barcelona, Spain; ^3^ Hepatobiliopancreatic Surgery and Abdominal Transplant Unit, Department of General Surgery, Hospital General Plaza de la Salud, Santo Domingo, Dominican Republic; ^4^ Barcelona Clinical Coordinating Center (BCCC) - Fundació Món Clínic, Barcelona, Spain; ^5^ Experimental Pathology Department, Institut d’Investigacions Biomèdiques de Barcelona-Consejo Superior de Investigaciones científicas (IIBB-CSIC), Barcelona, Spain; ^6^ Radiology Department, Hospital Clínic Barcelona, Barcelona, Spain; ^7^ Kidney Transplant Unit, Nephrology and Kidney Transplantation Department, Hospital Clinic Barcelona, Barcelona, Spain; ^8^ August Pi i Sunyer Biomedical, Research Institute (IDIBAPS), Barcelona, Spain; ^9^ Diabetes Unit, Endocrinology and Nutrition Department, Hospital Clínic Barcelona, Barcelona, Spain; ^10^ Network for Biomedical Research in Hepatic and Digestive Diseases (CIBERehd), Barcelona, Spain

**Keywords:** pancreas transplantation, graft survival, exocrine drainage, duodenoduodenostomy, intestinal complications

## Abstract

Techniques such as retroperitoneal graft placement have further enhanced the ability to replicate the physiology of the “native” pancreas. In our center, from January 2000, duodenojejunostomy (DJ) was the standard technique for exocrine drainage (n = 337). Herein, we report a series of 188 pancreas transplantations performed between May 2016 to July 2025, using a fully retrocolic graft position, systemic venous drainage and enteric drainage via duodenoduodenostomy. The primary endpoint was the assessment of intestinal events and their impact on graft and patient survival. A total of 14 patients (7.4%) experienced complications, including paralytic ileus (n = 2), intestinal obstruction (n = 4), duodenal dehiscence following pancreas transplantectomy (n = 1), anastomotic dehiscence (n = 5), and anastomotic bleeding (n = 2). Of these, 11 cases required relaparotomy for adhesiolysis (n = 2), internal hernia repair (n = 1), Hartmann’s procedure (n = 1), transplantectomy (n = 2), primary leak closure (n = 3), and hemostasis with duodenal re-anastomosis (n = 2). After a median follow-up of 42.8 months [IQR 21.8–71.1], graft survival at 1 and 5 years was 87% and 83.4%, respectively (P = 0.688 vs. DJ group), while patient survival was 100% and 98.2% (P = 0.031 vs. DJ group). Duodenoduodenostomy proved to be a feasible and effective technique, offering competitive outcomes in terms of graft and patient survival.

## Graphical Abstract

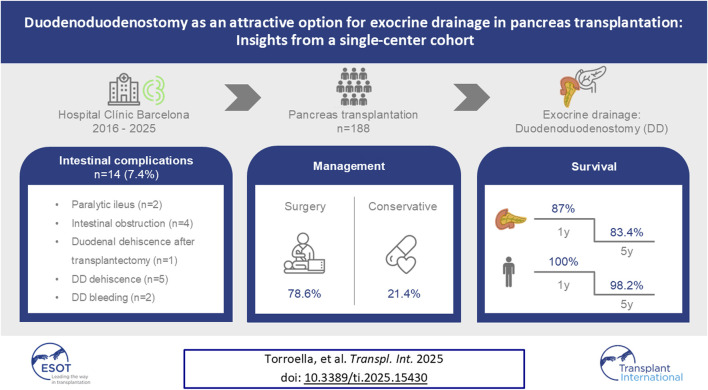

## Introduction

Pancreas transplantation has demonstrated its effectiveness in achieving normoglycemia and insulin independence, while also reducing metabolic instability and enhancing quality of life, with favorable long-term outcomes [1–3]. Since the first procedure in 1966, numerous refinements have been introduced, particularly concerning exocrine drainage, aiming to minimize morbidity and optimize results [4]. Initially, bladder drainage was the preferred method due to its utility in diagnosing rejection through urinary amylase monitoring [5]. However, its high complication rates and a substantial conversion rate to enteric drainage, reported to reach up to 40%, ultimately led to its decline [6, 7]. Today, enteric drainage is widely adopted, offering a more physiological approach and excellent clinical performance. Within this modality, several techniques have been developed, involving different segments of the small intestine. Two approaches have gained prominence: Roux-en-Y diversion and direct anastomosis to the jejunum or proximal ileal loop [8–10]. Additionally, alternative methods such as gastric anastomosis have also been described [11].

Although enteric diversion offers notable advantages, it is not without risks, presenting a considerable incidence of intra-abdominal adverse events and posing technical challenges in accessing the anastomosis and allograft for diagnostic and therapeutic purposes [1, 4].

Currently, various transplant centers have begun adopting direct duodenoduodenostomy (DD) as an alternative method for exocrine drainage, first introduced in 2007 [12]. In efforts to assess whether this approach offers improved graft survival and reduced postoperative morbidity, some case series have been published in recent years [13–23]. The benefits of endoscopic access for both diagnostic and therapeutic management have been highlighted, although interpreting duodenal biopsies remains challenging [24–27].

In line with the pursuit of safer and more reproducible surgical techniques that facilitate management of postoperative events, our team transitioned from duodenojejunostomy (DJ) to DD. This study aims to analyze the outcomes associated with DD, based on data collected from its implementation, focusing on two objectives. First, to assess intestinal events and their influence on graft and patient survival. Second, to compare these findings with the previously published historical series of DJ procedures performed at the same center, in order to evaluate the most appropriate surgical strategy for exocrine drainage moving forward.

## Materials and Methods

### Study Design

Pancreas transplants performed at the Hospital Clínic of Barcelona since the inception of the program in 1989 have been recorded in a prospectively maintained database.

In May 2016, direct DD was adopted as the preferred method for managing the exocrine output of the graft. All procedures carried out from that date through July 2025 were subsequently analyzed. These findings were then compared with the outcomes of the previously published series using DJ for enteric diversion, performed between 2000 and April 2016 at the same institution [28].

All categories of pancreas transplantation were included in the analysis: simultaneous pancreas-kidney (SPK), pancreas after kidney (PAK), pancreas alone (PA), and retransplantation. Recipients underwent a standardized preoperative evaluation conducted by a multidisciplinary team, following institutional criteria for indications and contraindications previously published [29]. Donor selection adhered to the consensus guidelines for pancreas and islet transplantation established by the National Transplant Organization [30].

### Prophylactic Therapy

Antibiotic prophylaxis consisted of ertapenem, vancomycin, and fluconazole, along with antiviral coverage for cytomegalovirus. Thromboprophylaxis was maintained using low molecular weight heparin and acetylsalicylic acid, following the protocol previously published elsewhere [28].

### Immunosuppression Regimens

Routine immunosuppression followed institutional protocols, with regimens varying according to the date of transplantation [31]. During the SARS-CoV-2 pandemic, adjustments were made between April 2020 and May 2021 due to increased immunological risk. During this period, induction therapy with the anti-interleukin-2 monoclonal antibody (basiliximab) was preferred over rabbit anti-human polyclonal antibodies (thymoglobulin).

### Surgical Technique

The pancreas is harvested *en bloc* along with the donor duodenum and spleen, and the graft is subsequently prepared on the back table. Arterial reconstruction is performed either by (a) end-to-end anastomosis between the splenic artery and the distal superior mesenteric artery, or (b) reconstruction with an iliac arterial “Y” graft. Additionally, the donor duodenum is transected with a mechanical stapler to a length of approximately 8–10 cm, and the edges are reinforced with either continuous or interrupted hand-sewn sutures using 3-0 non-absorbable material [32].

**FIGURE 1:**
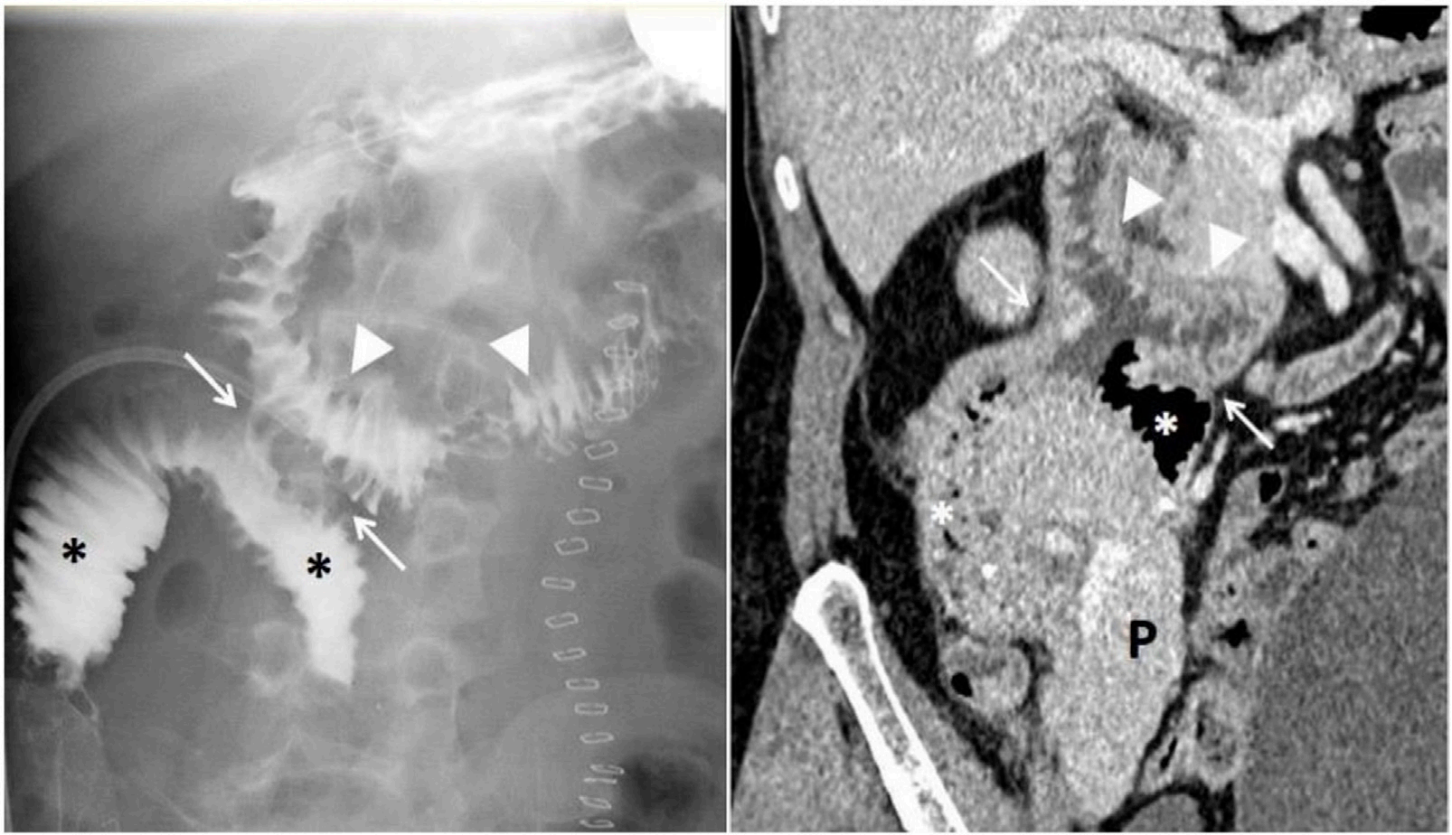
Pancreas transplantation with retrocolic graft placement and duodenoduodenostomy for exocrine drainage. Esophagogastroduodenal transit study with Gastrografin (right) demonstrates proper contrast passage through the recipient’s duodenum (arrowheads), the side-to-side duodenoduodenal anastomosis (white arrows), and the graft’s duodenal segment (asterisks), with no evidence of leakage. Abdominal contrast-enhanced computed tomography of the same patient (left) shows a normoenhancing pancreatic graft (P), without collections or other complications.

**FIGURE 2:**
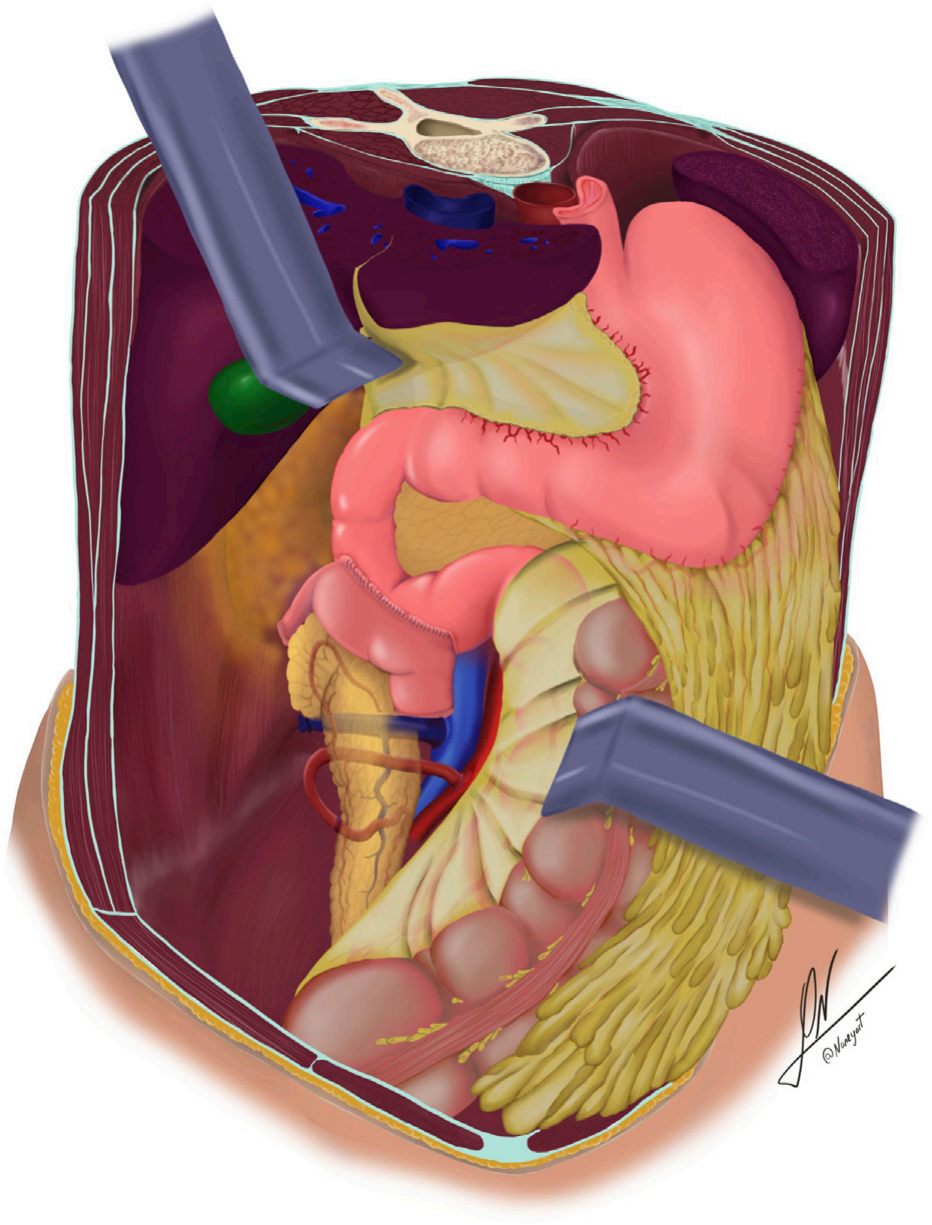
Schematic illustration showing retrocolic placement of the pancreatic graft, systemic venous drainage, and enteric drainage via side-to-side duodenoduodenostomy.

Recipient surgery was performed via midline laparotomy. Graft placement varied depending on the study period: (a) in the DD group, the pancreas was positioned retrocolically on the right side of the pelvis following mobilization of the right colon using the Cattell-Braasch and Kocher maneuvers, with the duodenal segment oriented cephalad [21] (Figures 1, 2); or (b) in the DJ group, the graft was placed intraperitoneally, as previously described [28]. Venous drainage was directed into the systemic circulation through an anastomosis between the graft’s portal vein and either the inferior vena cava or the recipient’s right iliac vein. Arterial supply was established via an end-to-side anastomosis between the graft’s superior mesenteric artery, or the Y-graft, depending on the reconstruction technique used during back-table preparation, and the recipient’s common right iliac artery.

**FIGURE 3:**
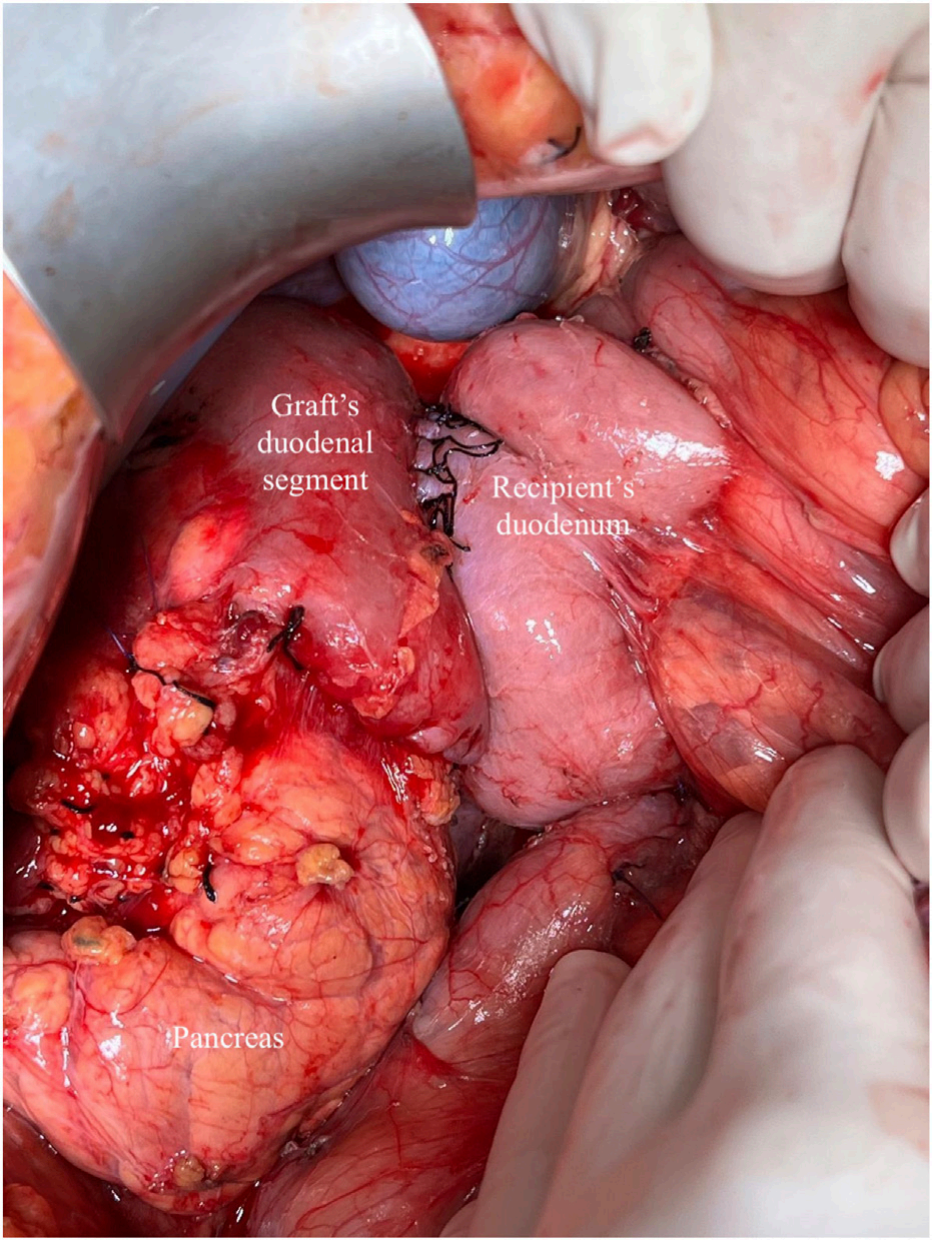
Whole-Organ transplant with systemic venous and enteric exocrine drainage (cephalad position). Duodenoduodenostomy technique with side-to-side anastomosis between the duodenal segment and lower knee of the recipient’s duodenum.

**FIGURE 4:**
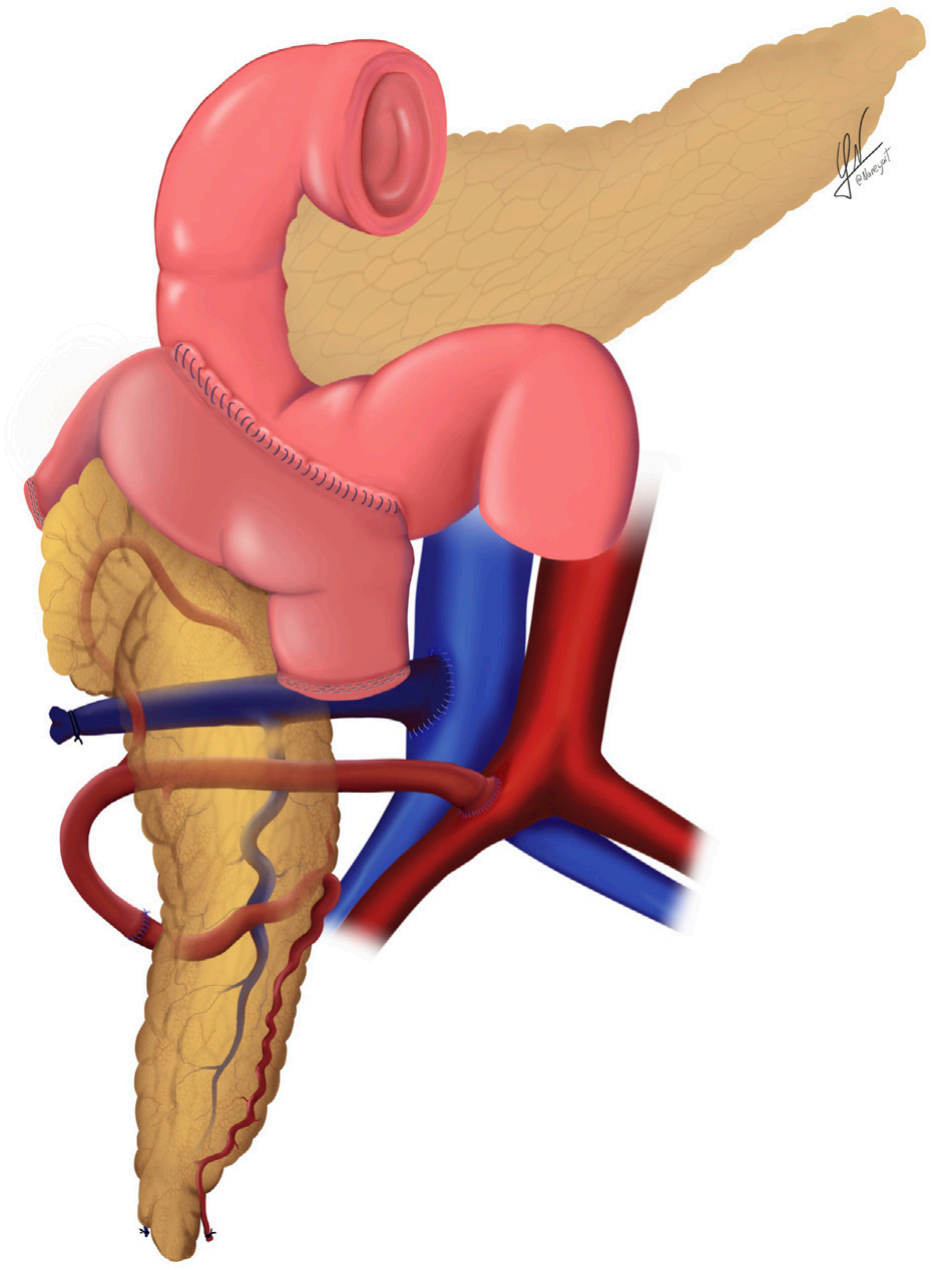
Schematic illustration of the systemic venous drainage technique combined with duodenal enteric drainage.

Following graft revascularization and achievement of haemostasis, the enteric anastomosis is performed. In the DD group, a side-to-side hand-sewn anastomosis is constructed using a double-layer technique: the outer layer with non-absorbable 3-0 sutures and the inner layer with absorbable 3-0 sutures, applied either continuously or with interrupted stitches (Figures 3, 4). In contrast, the DJ group underwent a side-to-side anastomosis without Roux-en-Y, positioned 60–80 cm distal to the ligament of Treitz. This was also performed using a double-layer hand-sewn technique, employing 3-0 non-absorbable sutures for the external layer and 3-0 absorbable sutures for the inner layer, as previously described [28].

### Patient Progress and Follow-Up

Postoperative morbidity was defined as any complication occurring within 90 days following pancreas transplantation, and was categorized according to the Clavien-Dindo classification system [33]. Diagnostic approaches were also assessed. Doppler ultrasound (US) is the standard imaging technique used in the postoperative period. A mandatory US is performed within 24–48 h after transplantation to evaluate vascular patency, graft integrity, and potential indirect signs of intra-abdominal complications. In cases presenting with acute abdominal symptoms, graft dysfunction, or inconclusive ultrasound results, computed tomography or Angio-CT was employed. When acute rejection was suspected, based on biochemical markers, imaging findings, or poor glycemic control, graft biopsies were obtained. Rejection episodes were classified according to the Banff criteria [34].

Management of intestinal complications was tailored to the patient’s clinical condition and the severity of the adverse event.

### Statistical Analysis

Categorical variables are presented as absolute frequencies and percentages (%). Quantitative variables are expressed as median and interquartile range [IQR] (25th–75th percentiles). Categorical variables were analyzed by use of Fisher’s exact or chi-square test, and continuous variables were analyzed by unpaired Student’s t-test, Mann–Whitney U-test, or other nonparametric tests.

Pancreas graft survival was calculated from the time of transplant until the return to permanent insulin therapy dependency, or death/end of follow-up with a functioning graft. Patient survival was calculated from the time of transplant to death or the end of follow-up.

Patient and graft survival rates were compared between exocrine drainage technique groups by means of Kaplan-Meier curves and the Log-rank test. A Cox proportional hazards model was used to obtain a hazard ratio estimate for DD vs. DJ adjusting for the potential confounders of technique effects, i.e., those factors showing a clinically and statistically significant association with the technique group (Table 1) and with 10-year mortality (Supplementary Table S1) or 10-year graft survival (Supplementary Table S2).

**TABLE 1:** Donor and recipient characteristics.

	Total (n = 525)	DD group (n = 188)	DJ group (n = 337)	*P* Value
**DONOR**				
Type of donor - DBD - cDCD III	493 (93.9%) 32 (6.1%)	156 (83%) 32 (17%)	337 (100%) -	<0.001^a^
Cause of death - Trauma - CVA - Anoxic damage - Euthanasia - Others	251 (47.8%) 207 (39.4%) 41 (7.8%) 4 (0.8%) 22 (4.2%)	69 (36.7%) 86 (45.7%) 22 (11.7%) 4 (2.1%) 7 (3.7)	182 (54%) 121 (35.9%) 19 (5.6%) - 15 (4.5%)	<0.001^b^
Age (years)	33 [21–42]	36 [23–46]	31 [21–40]	0.0004^c^
Gender (M/F)	313 (59.6%)/212 (40.4%)	107 (56.9%)/81 (43.1%)	206 (61.1%)/131 (38.9%)	0.345^b^
BMI (Kg/m^2^)	23.4 [21.5–25.3]	23.5 [21.6–25.1]	23.4 [21.6–25.4]	0.583^c^
ICU stay (days)	2 [1–5]	2 [1–5]	3 [1.7–7]	0.242^d^
Amylase (IU/L)	80 [47–156.5]	75 [41–133]	84 [48.8–170]	0.059^d^
Lipase (IU/L)	34.5 [17–95.2]	31 [16–64]	45 [17.5–115]	0.079^d^
P-PASS total	16 [14–18]	17 [15.2–18]	16 [14–18]	0.208^c^
Preservation solution - UW - CS - HTK - IGL-1	271 (51.6%) 99 (18.9%) 8 (1.5%) 147 (28%)	15 (8%) 32 (17%) 1 (0.5%) 140 (74.5%)	256 (76%) 67 (19.9%) 7 (2.1%) 7 (2.1%)	<0.001^b^
Pancreas CIT^e^ (hours)	10 [7–11.5]	7.5 [6–10]	10.3 [8–12]	<0.001^c^
**RECIPIENT**				
Age (years)	41 [35–47]	43 [36–49]	40 [35–45]	<0.001^a^
Gender (M/F)	330 (62.9%)/195 (37.1%)	110 (58.5%)/78 (41.5%)	220 (63.3%)/117 (34.7%)	0.123^b^
BMI (Kg/m^2^)	23 [21–25.6]	23 [21.1–25.3]	23 [20.8–25.9]	0.799^a^
Type of DM - DM 1 - DM 2 - Others	516 (98.3%) 3 (0.6%) 6 (1.1%)	183 (97.3%) 3 (1.6%) 2 (1.1%)	333 (98.8%) - 4 (1.2%)	0.067^b^
DM *vintage* (years)	26 [21–32]	28 [22–34]	26 [ 21–31]	0.011^c^
Dialysis *vintage* (months)	24 [13.9–35.7]	18 [11–27]	27.0 [19.4–37.7]	<0.001^c^
Type of dialysis - Predialysis - Peritoneal - Hemodialysis - None	63 (12%) 108 (20.6%) 291 (55.4) 63 (12%)	36 (19.1%) 31 (16.5%) 103 (54.8%) 18 (9.6%)	27 (8%) 77 (22.8%) 188 (55.8%) 45 (13.4%)	<0.001^b^
Transplant type - SPK - PAK - PA - Retransplant	444 (84.6%) 30 (5.7%) 3 (0.6%) 48 (9.1%)	168 (89.4%) 7 (3.7%) - 13 (6.9%)	276 (81.9%) 23 (6.8%) 3 (0.9%) 35 (10.4%)	0.107^b^

^a^ Fisher Exact p-value.

^b^ Chi-Square p-value.

^c^ Equal variance two sample t-test.

^d^ Kruskal-Wallis p-value.

Continuous variables are expressed as median [interquartile ranges (IQR)] and categorical variables as frequencies (percentages).

BMI, body mass index; cDCD, controlled donation after circulatory death; CS, celsior; CIT, cold ischemia time; CVA, cerebrovascular accident; DBD, donation after brain death; DD, duodenoduodenostomy; DJ, duodenojejunostomy; DM, diabetes mellitus; F, female; HTK, Histidine-Tryptophan-Ketoglutarate; IGL-1, Institut Georges Lopez-1; ICU, intensive care unit; M, male; PPASS, preprocurement pancreas suitability score; PAK, pancreas after kidney; PA, pancreas transplant alone; SPK, Simultaneous Pancreas-Kidney; UW, university of wisconsin.

^e^ CIT, is the interval between the initiation of organ perfusion with cold preservation solution in the donor and the onset of reperfusion in the recipient.

A P value <0.05 was considered to indicate statistical significance. Data are collected and analyzed with SPSS statistical software (SPSS 20.0, 1989-1995; Chicago, IL, United States) and SAS v 9.4 software (SAS Institute, Cary, North Carolina, United States).

## Results

### Donor and Recipient Demographic Data

Between January 2000 and July 2025, a total of 526 pancreas transplants were performed in our unit. From January 2000 to April 2016, DJ was the technique employed for exocrine drainage (n = 337). In May 2016, a transition to DD was implemented, with 188 transplants performed to date, which constitute the focus of this study. It is noteworthy that, in one case (excluded from the analysis) during this period, a DJ was performed due to a technical challenge, as the inferior vena cava was positioned to the left of the aorta.

The characteristics of the donors and recipients are summarized in Table 1. Historically, all donors were donation after brain death (DBD). Controlled donation after circulatory death (cDCD) with normothermic regional perfusion was introduced at our center in 2019, with 32 cases performed to date, all within the DD group. The median donor age was significantly higher in the DD group (P < 0.001), with cerebrovascular disease being the leading cause of death (45.7%). In the DJ group, donor amylase and lipase levels were substantially elevated, with a trend toward statistical significance (P = 0.059 and P = 0.079, respectively). No significant differences were observed between groups regarding gender, body mass index (BMI), ICU stay duration, or Pancreas Donor Risk Index (P-PASS). Preservation solutions varied across groups and time periods. Institut Georges Lopez-1 (IGL-1) was predominantly used in the DD group (74.5%) following its introduction at our center in 2015 (31), whereas the University of Wisconsin (UW) solution was employed in 76% of DJ cases (P < 0.001). Cold ischemia time (CIT) for the pancreas was significantly shorter in the DD group [7.5 h (IQR 6–10)] compared to the DJ group [10.3 h (IQR 8–12)] (P < 0.001).

As for recipient demographics, age was slightly higher in the DD group (P < 0.001), with a predominance of male patients. Notably, the DD group exhibited a shorter duration of dialysis prior to transplantation (P < 0.001) and a longer history of diabetes mellitus (P = 0.011). Previous abdominal surgery was documented in nearly 40% of patients in the DD group. SPK transplantation was the most frequently performed procedure in both groups. For other transplant categories (PAK, PA, and retransplantation) the majority of cases belonged to the DJ group. Backtable vascular graft reconstruction using a splenic artery-superior mesenteric artery technique was performed in 85.6% of DD cases and 95.7% of DJ cases, with the remaining procedures utilizing a Y-graft configuration.

Post-reperfusion serum levels of amylase and lipase (measured at 24-48 h) were also analyzed. Amylase levels showed no significant difference between groups [DD: 168 (IQR 114.5–272.5) vs. DJ: 195 (IQR 111–349); P = 0.176], while lipase levels were significantly lower in the DD group [DD: 154.5 (IQR 84–268.5) vs. DJ: 183.5 (IQR 100–389.2); P = 0.041].

### Postoperative Outcomes

Intestinal complications, a central focus of this study, were identified in 14 patients within the DD group (7.4%) (Table 2). Both mild and severe cases were included, encompassing patients managed conservatively as well as those requiring more intensive interventions. All diagnoses were confirmed through radiological imaging, specifically computed tomography scan.

**TABLE 2:** Summary of intestinal complications.

Transplant type (year)	Donor type/cause of death Gender/age (years)	CIT (hours)	Intestinal complication	Recipient gender/age (years)/BMI (Kg/m^2^)	Treatment Post-transplant day of treatment	Graft/patient survival (months)
SPK (2018)	DBD/CVA Male/24	11.10	Intestinal obstruction *No vascular event* Clavien-Dindo: IIIb	Male/36/18.7	Surgery: Internal hernia repair (15° day)	85.8/85.8 Alive
Pancreas Retx (2018)	DBD/CVA Female/38	11.30	Recipient duodenum dehiscence after transplantectomy (*vascular thrombosis*) Clavien-Dindo: IIIa	Female/57/21.3	Endoscopic clip (72° day)	1.18/79.8 Alive
SPK (2019)	DBD/CVA Female/41	12	DD dehiscence *No vascular event* Clavien-Dindo: IIIb	Male/53/33.1	Transplantectomy (8° day)	0.85/18.6 Death (sepsis)
SPK (2019)	DBD/Trauma Male/36	9	Paralytic ileus V*enous (SMV) peripheral thrombosis* ^a^ Clavien-Dindo: II	Female/43/24.9	Nasogastric tube TPN (8° day)	44.2/44.2 Death (Brest cancer)
SPK (2019)	DBD/CVA Female/56	6	DD dehiscence *Distal arterial thrombosis head of pancreas* ^a^ Clavien-Dindo: IIIb	Male/42/22	Primary leak closure (11° day)	68.6/68.6 Alive
SPK (2020)	DBD/CVA Male/20	7.40	Intestinal obstruction *No vascular event* Clavien-Dindo: IIIb	Male/46/21.2	Adhesiolysis between the colon and pancreatic tail (12° day)	62.9/62.9 Alive
SPK (2020)	DBD/Trauma Male/24	12	Paralytic ileus V*enous (SV) peripheral thrombosis* ^a^ Clavien-Dindo: II	Male/56/31.8	Nasogastric tube TPN (7° day)	56.2/56.2 Alive
SPK (2021)	DBD/Trauma Male/18	10.6	Intraluminal DD bleeding *No vascular event* Clavien-Dindo: IIIb	Female/45/23.9	Hemostasis and DD re-anastomosis (11° day)	16.6/43.9 Chronic rejection Alive
SPK (2022)	cDCD/Neurodegenerative disorder Male/46	4.2	DD dehiscence Hemoperitoneum (1° day: reoperation) *No vascular event* Clavien-Dindo: IIIb	Male/43/27	Primary leak closure (7° day)	41.2/41.2 Alive
SPK (2022)	DBD/CVA Male/48	5.6	Intestinal obstruction 2° to pancreatitis *No vascular event* Clavien-Dindo: IIIb	Male/46/25.9	Adhesiolysis (11° day)	0.36/40.2 Primary Graft Dysfunction Alive
PAK (2022)	cDCD/CVA Male/46	4	Intestinal obstruction 2° to stercoral colitis Clavien-Dindo: IIIb	Male/45/25.2	Hartmann’s procedure (37° day)	34.5/34.5 Alive
SPK (2022)	DBD/Trauma Male/40	11	Anastomotic bleeding V*enous (SV and SM) occlusive thrombosis* Clavien-Dindo: IIIb	Female/30/31.2	Thrombectomy and hemostasis and DD re-anastomosis (6° day)	33.7/33.7 Alive
SPK (2022)	DBD/CVA Female/50	5.2	DD dehiscence *No vascular event* Clavien-Dindo: IIIb	Male/46/21.6	Primary leak closure (15° day)	32.4/32.4 Alive
SPK (2024)	cDCD/Anoxia Male/36	5.2	DD dehiscence V*enous (SV and SM) occlusive thrombosis* Clavien-Dindo: IIIb	Male/42/29.7	Transplantectomy (7° day)	0.3/17.2 Alive

BMI, body mass index; cDCD, controlled donation after circulatory death; CIT, cold ischemia time; CVA, cerebrovascular accident; DBD, donation after brain death; DD, duodenoduodenostomy; PAK, pancreas after kidney; Retx, pancreas retransplantation; SPK, Simultaneous Pancreas-Kidney; SMV, superior mesenteric vein; SV, splenic vein; TPN, total parenteral nutrition.

^a^ Anticoagulation protocol [Low molecular weight heparin (20 mg/12 h), and acetylsalicylic acid (50 mg/24 h)].

Two patients developed *postoperative paralytic ileus*, both successfully managed with a conservative approach involving nasogastric decompression and total parenteral nutrition. In both cases, peripheral venous thrombosis was also identified during routine US follow-up. This finding did not affect pancreatic graft function and did not necessitate any modification to the standard anticoagulation protocol, which consists of low molecular weight heparin at a dose of 20 mg every 12 h, combined with acetylsalicylic acid at 50 mg once daily.

Four patients experienced *intestinal obstruction*. In two cases, the cause was adhesions involving the colon and small intestine secondary to graft pancreatitis, requiring reoperation for adhesiolysis. Another patient presented with an internal hernia, which also necessitated surgical intervention. Lastly, a Hartmann’s procedure was performed in a patient diagnosed with stercoral colitis on postoperative day 37. None of these cases were associated with vascular events.

A *recipient duodenal leak* following graft transplantectomy due to venous thrombosis was also observed. The diagnosis was made based on the bilious appearance of the abdominal drainage. The complication was managed endoscopically and successfully sealed using the OVESCO system (OTSC^®^, Ovesco Endoscopy GmbH, Tübingen, Germany), with satisfactory clinical outcomes.

*Anastomotic duodeno-duodenostomy dehiscence* was diagnosed in five patients. One presented with abdominal pain, while the remaining cases were identified due to bilious output from abdominal drainage. Three patients underwent reoperation, with successful primary closure of the infracentimetric leak and reinforcement of the anastomosis using separate stitches, resulting in good postoperative recovery and preserved pancreatic graft function. The remaining two cases had previously undergone unsuccessful surgical attempts to repair the leak, ultimately requiring graft removal. One recipient had a BMI of 33 and a CIT of 12 h, with evidence of duodenal hypoperfusion observed at the time of reperfusion. The second case involved a marginal cDCD donor who had been supported with ECMO for 12 days and also presented with associated occlusive vascular thrombosis.

*Bleeding from the duodeno-duodenostomy* anastomosis was identified in two patients, prompting surgical intervention to revise the intestinal anastomosis and achieve duodenal hemostasis. Notably, one of these cases also presented with venous occlusive thrombosis, for which a thrombectomy was performed. The graft was successfully salvaged and remains functional at 33.7 months post-transplantation.

Overall, in the DD group, the median length of hospital stay was 12 days [IQR 10–20]. Patients who experienced intestinal complications had significantly longer hospitalizations, with a median of 27.5 days [IQR 16–38.5] (P < 0.001).

### Patient and Graft Survival

**FIGURE 5:**
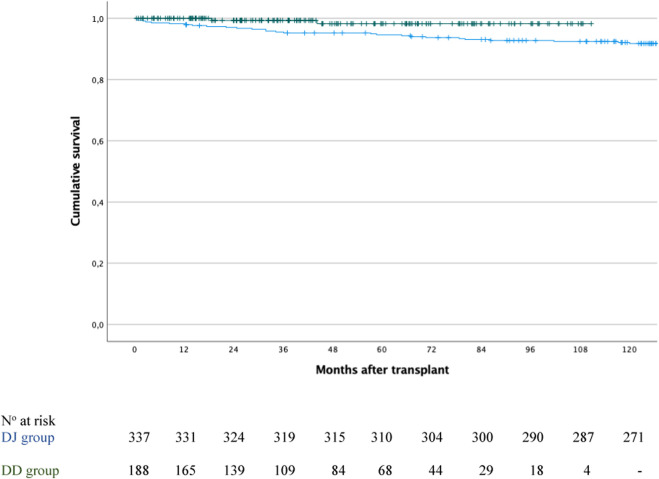
Patient survival in the duodenoduodenostomy group (green line) versus the duodenojejunostomy group (blue line); P = 0.031.

After a median follow-up of 124.9 months [IQR: 49.6–205.1] for the entire cohort (n = 525), overall patient survival at 1, 3, 5, and 10 years was 98.8%, 96.7%, 95.8%, and 93.3%, respectively. When stratified by group, survival rates were 100%, 99.3%, 98.2% and 97.6% for the DD group, and 98.2%, 95.5%, 94.6% and 92.1% for the DJ group (P = 0.031; Figure 5).

Focusing on the DD group, after a median follow-up of 42.8 months [IQR 21.8–71.1], patient survival was 100% in both the intestinal complication and non-complication groups at 1 year. At 3 years, survival was 92.3% in the complication group versus 100% in the non-complication group, and at 5 and 10 years, 76.9% versus 100%, respectively (P < 0.001). Two patients died: one due to abdominal sepsis at 18.6 months post-transplant, and another from metastatic breast cancer at 44.2 months post-transplant.

**FIGURE 6:**
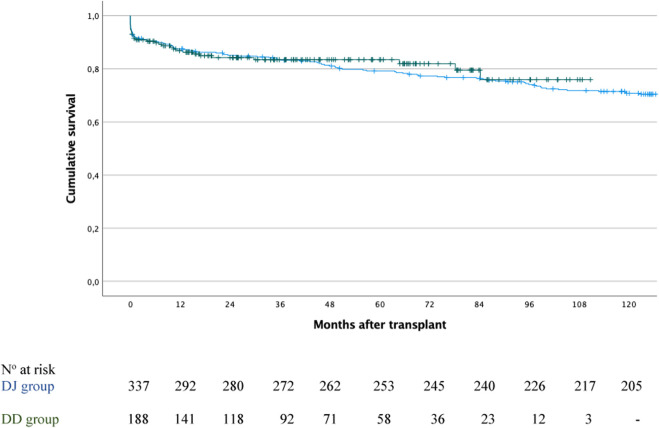
Pancreas graft survival in the duodenoduodenostomy group (green line) versus the duodenojejunostomy group (blue line); P = 0.688.

Furthermore, among the 525 patients, death-censored pancreas graft survival at 1, 3, 5, and 10 years was 87.3%, 83.4%, 80% and 71.3%, respectively. When stratified by group, survival rates were 87%, 83.4%, 83.4% and 75.9% for the DD group, and 87.5%, 83.5%, 79.1% and 70.8% for the DJ group (P = 0.688; Figure 6).

In the DD group, graft survival was significantly lower in patients with intestinal complications compared to those without: 71.4% versus 88.1% at 1 year, 64.3% versus 85% at 3 years and 5 years, and 64.3% versus 77.1% at 10 years (P = 0.049).

After adjusting the effect of surgical technique on patient survival for the potential confounders observed in Table 1 and in Supplementary Table S1 (donor age, amylase levels and pancreas CIT), the estimated risk for DD vs. DJ was a hazard ratio (HR) of 0.47 (95% CI 0.10–2.14; P = 0.3264).

The Cox proportional hazards model fitted for 10-year graft survival adjusted group effects for recipient age, type of dialysis, and for donor pancreas CIT. The estimated HR for DD vs. DJ was 1.05 (95% CI 0.67–1.64; P = 0.8378).

## Discussion

Exocrine drainage has undergone several variations throughout history. Bladder drainage, which was predominant until the mid 90s, allows for immunological monitoring of the graft through the determination of pancreatic enzymes in urine or by cystoscopic biopsy [5, 35]. However, reflux pancreatitis and urinary bicarbonate losses causing metabolic acidosis and dehydration led to its replacement by enteric drainage. Despite technical advances, no universally accepted surgical approach has been established to date. The choice of technique varies significantly across transplant centers, influenced by team experience, recipient characteristics, and institutional preferences. This heterogeneity reflects both the complexity of the procedure and the need to tailor treatment to the clinical context. Traditionally, enteric anastomosis can be performed at the level of the proximal jejunum or distal ileum, either end-to-end, end-to-side, or side-to-side. Although Boggi et al [8] described excellent results using the Roux-en-Y side-to-side DJ, the direct side-to-side duodenojejunostomy is more prevalent [10, 28].

An attractive option arises when the pancreas is placed in the right retrocolic position, the direct side-to-side DD, which allows for a more physiological graft placement. The main advantage of DD is its endoscopic accessibility [24–27], as it facilitates pancreatic and duodenal biopsies for the diagnosis of rejection and allows for stenting of the pancreatic duct in cases of pancreatic fistulas.

A key element promoting the use of DD was its role in pancreas alone transplantation, which faces a higher risk of immunological graft loss compared to SPK transplantations due to the absence of monitoring tools for rejection without a sentinel kidney graft. Consequently, the endoscopic accessibility to the graft duodenum was a significant factor that set off the expansion of DD worldwide.

Beyond this potential advantage, parallel considerations must be taken into account by the fact that retrocolic placement of the graft raised concerns regarding restricted access for percutaneous biopsy, potentially complicating postoperative monitoring. However, recently, Bassaganyas et al. [36] demonstrated that percutaneous biopsy accessibility in retrocolic positioned grafts implanted via duodenoduodenal anastomosis is not only feasible but also associated with a high success rate in terms of histological diagnosis. Moreover, the accessibility rate was found to be even higher than that observed in intraperitoneally positioned grafts (87.9% in DD group compared to 68.4% in DJ group). These findings challenge the previously held perception that retrocolic positioning might hinder access and instead support the use of this technique as a safe and effective tool for the post-transplant follow-up of pancreatic grafts. Due to the favorable outcomes achieved with percutaneous biopsy at our center, transduodenal biopsies have not been required thus far. Nonetheless, this does not exclude the possibility that such procedures may be necessary in future cases.

The first case reports performing DD were published in 2007 [13] and since then, many centers have adopted this technique [37]. In 2012, Gunasekaran et al [17] presented the first series of 21 cases of DD and compared its results with DJ, not finding significant differences between groups in terms of graft survival. In 2014, the German group from Bochum [18] published one of the largest series to date with 125 cases of DD. Survival rates were slightly better in the DD group than DJ group, although this difference was not statistically significant (p = 0.202). Graft thrombosis, anastomotic insufficiency, and the need for relaparotomy occurred less frequently in the DD group. These findings may be attributed to the relatively fixed nature of the anastomosis to the duodenum, which likely reduces the risk of graft vessel torsion or twisting. In the same year, Perosa et al. [20] described their first experience with 53 cases of DD. To date, the Brazilian group represents the largest reported Latin American series involving the duodenal drainage technique, comprising a total of 248 cases [38]. With over 25 years of experience and more than 1,000 transplants performed, the authors consider that combining DD anastomosis with venous drainage to the inferior vena cava, particularly when using a very short graft vein, constitutes an optimal surgical strategy. In fact, this is the technique currently employed at our center [21]. This configuration has been associated with low rates of thrombosis and anastomotic leakage, while preserving the advantages of easy access to the pancreatic graft [39].

Nevertheless, some transplant centers have discontinued the routine use of DD due to its limitations in clinical practice. The Norwegian group [40] reported low sensibility of duodenal graft biopsies for detecting pancreas graft rejection, while Ryu et al. [41], representing the Korean group, highlighted the greater technical complexity of DD compared to DJ. Notably, Ryu’s team performed the first DD in South Korea and recently published their experience with 100 consecutive pancreas transplants, of which 86 were performed using DD and 14 using DJ. Their analysis reported a remarkably low incidence of surgical complications, including adhesions, technical failure, duodenal graft leakage, and vascular thrombosis, along with pancreatic graft survival rates of 91% at 1 year and 78.5% at 5 years. Despite these favorable outcomes, the group transitioned back to DJ, discontinuing routine use of DD in mid-2022 due to its greater technical complexity.

Our group reported the initial series of 10 cases employing duodenal drainage in 2017 [21], with no complications linked to the innovative surgical approach. These promising results laid the groundwork for its consolidation. Since then, DD has become the preferred method for enteric drainage.

In the current cohort of 188 procedures, an intestinal complication rate of 7.4% (n = 14) was observed, which is comparable to the rate reported in our historical series using DJ (6.8%; p = 0.789) [28].

Five cases of anastomotic dehiscence were recorded, with two requiring transplantectomy, likely due to impaired duodenal vascularization. Vascular events were identified in two of the three cases, including distal arterial thrombosis and venous occlusion, the latter ultimately leading to graft removal.

Potential disadvantages of DD include the difficulty of duodenal repair in the event of anastomotic dehiscence. Nevertheless, the most experienced groups in this type of anastomosis agree that DD is a safe procedure, with no significant increase in the incidence of intestinal complications [37]. In fact, it has been suggested that the retrocolic positioning of the graft may reduce the risk of peritonitis in the event of an intestinal leak, by limiting the intraperitoneal spread of duodenal contents [17, 23]. This observation aligns with the fact that, in three cases, DD dehiscence was successfully managed by primary closure while preserving graft function, a maneuver that, in the presence of peritonitis, would have posed greater technical challenges and likely resulted in less favorable outcomes. In the DJ cohort [28], 2 out of 4 cases of duodenum-jejunum anastomotic dehiscence required pancreas removal. Additional events compromising grafted duodenum integrity were also documented. Among these, three patients developed intestinal fistulas originating from the duodenal edge, without anastomotic failure; two underwent transplantectomy within 15 days post-surgery. Duodenal ischemia proved devastating in two cases, both necessitating graft excision. Furthermore, one individual required reoperation due to duodenal graft torsion, which was corrected by repositioning the graft medially. It is worth noting that intraperitoneal placement of the pancreas may facilitate twisting, thereby increasing the likelihood of venous thrombosis. Duodenal dehiscence following transplantectomy was also reported in one patient, which was successfully treated with endoscopic clip placement. This case highlights one of the advantages of DD, as it allowed for a more conservative approach to the management of complications, thereby avoiding the need for a surgical intervention. In contrast, a patient with prior DJ who presented with anastomotic dehiscence in the recipient jejunum after graft transplantectomy, required a repeated laparotomy for resection of the affected intestinal segment [28]. An additional benefit observed with DD is the low incidence of paralytic ileus, which occurred in only two patients in our series and was successfully managed conservatively. As previously noted, the retrocolic positioning of the graft contributes to this reduced rate, as ischemia-reperfusion–induced pancreatitis remains confined to the retroperitoneal space [17, 23]. This observation is further supported by comparison with the 21.7% incidence reported in our series of patients who received DJ [28], a figure that may in fact be underestimated due to the inclusion of individuals transplanted more than two decades ago. Four cases of intestinal obstruction were identified, although only two were attributed to postoperative adhesions. Additionally, one patient developed stercoral colitis, which necessitated a Hartmann’s procedure. Notably, only one case of internal hernia was documented. This low incidence may be attributed to the limited mobility of the graft in its retrocolic position, as previously described [18]. Interestingly, 7 out of 23 patients in the DJ group required surgery for adhesiolysis secondary to graft pancreatitis [28], a significantly higher proportion. A manual suture was employed in all DD cases and anastomotic bleeding was observed in two cases, both requiring reconfection of the duodenoduodenal anastomosis. In one case, the bleeding was concomitant with venous occlusive thrombosis requiring an additional thrombectomy procedure. In contrast, Gunasekaran et al [17] initially hypothesized a higher incidence of anastomotic site bleeding in the DD group but subsequent analyses attributed this complication primarily to the use of surgical staplers rather than the anastomotic technique itself. Although they perform the intestinal suturing manually, the German group [18] reported that gastrointestinal bleeding complications occurred more frequently in the DD group (11%) compared to DJ group (3%). Half of these cases were successfully treated with endoscopic haemostasis, although 28% required surgical intervention.

Despite the theoretical advantages of DD over DJ, findings from our cohort suggest that both techniques yield comparable outcomes, with similar rates of intestinal complications and competitive graft and patient survival. Notably, overall patient survival in the DD group was significantly higher compared to the historical DJ cohort (P = 0.031). It is also important to acknowledge that data collection for the DD group was considerably more comprehensive, whereas the DJ series corresponds to procedures performed prior to 2016. This discrepancy may introduce a degree of bias in the comparative analysis between both cohorts.

It should be emphasized that, beyond the surgical technique itself, multiple factors may predispose to early postoperative intestinal complications. These include ischemia of the duodenal cuff, impaired tissue repair due to immunosuppressive therapy, trauma to the donor duodenum during procurement or back-table preparation, and intra-abdominal infections, among others. Additionally, donor-related variables and triggers of post-reperfusion pancreatitis must not be overlooked. In this regard, the DD group included donors considered marginal on paper, such as cDCD, which may partly explain the slightly lower graft survival observed during the first year compared to the DJ group (P = 0.688).

To improve outcomes, logistical optimizations have been implemented at our center. Specifically, for recipients with a panel-reactive antibody (PRA) of 0% (at least 2 determinations in the past 6 months), a virtual crossmatch is performed. Hence the surgical procedure commences without awaiting the Complement-Dependent Cytotoxicity (CDC) or flow cytometry crossmatch results, leading to a reduction in the CIT. Furthermore, the protocol for lymph node transport from external donor hospitals has been refined. Lymph nodes are now harvested at the beginning of the donor procedure and immediately transferred to our center, enabling crossmatch testing to be completed prior to organ arrival.

To our knowledge, this constitutes one of the largest published series involving retrocolic graft positioning combined with DD for exocrine drainage. We deliberately focused on complications directly related to the DD itself, aiming to provide a targeted analysis within this specific area of study. Although the study is retrospective, single-center, and non-randomized, it benefits from the consistent application of clinical management protocols and a standardized surgical technique, which contribute to the homogeneity of the cohort.

Ultimately, DD represents a technically viable alternative in pancreas transplantation, with clinical outcomes comparable to other enteric drainage modalities. Its adoption broadens the spectrum of surgical options, particularly in retransplantation scenarios. Owing to its favorable technical profile and the added benefit of facilitating graft access for biopsy when needed, both endoscopic and percutaneous, duodenoduodenostomy stands out as a contemporary and relevant strategy within the array of available surgical approaches.

## Data Availability

The original contributions presented in the study are included in the article/Supplementary Material, further inquiries can be directed to the corresponding author.

## Abbreviations

BMI, Body mass index; CIT, Cold ischemia time; DBD, Donation after brain death; cDCD, Controlled donation after circulatory death; DD, Duodenoduodenostomy; DJ, Duodenojejunostomy; IGL-1, Institute Georges Lopez-1; PAK, Pancreas after kidney; PA, Pancreas alone; SPK, Simultaneous pancreas-kidney; US, Ultrasound; UW, University of Wisconsin.
