# Supplementary material for: Duodenoduodenostomy as an Attractive Option for Exocrine Drainage in Pancreas Transplantation: Insights From a Single-Center Cohort
Source: Transpl Int. 2025 Nov 3;38:15430. doi: 10.3389/ti.2025.15430 (PMC12620303; doi:10.3389/ti.2025.15430)
Supplement: Supplementary file 1 [file Table1.docx]

**Supplementary Table 1.** Mortality factors

|  | Total  (n = 525) | Survivor  (n = 495) | Death  (n = 30) | *P* value |
| --- | --- | --- | --- | --- |
| DONOR |  |  |  |  |
| Type of donor  - DBD  - cDCD III | 493 (93.9%)  32 (6.1%) | 463 (93.5%)  32 (6.5%) | 30 (100%)  - | 0.2446^1^ |
| Cause of death  - Trauma  - CVA  - Anoxic damage  - Euthanasia  - Others | 251 (47.8%)  207 (39.4%)  41 (7.8%)  4 (0.8%)  22 (4.2%) | 234 (47.3%)  200 (40.4%)  36 (7.3%)  4 (0.8%)  21 (4.2%) | 17 (56.7%)  7 (23.3%)  5 (16.7%)  -  1 (3.3%) | 0.1913^2^ |
| Age (years) | 33.0 (9.0, 59.0) | 33.0 (9.0, 59.0) | 23.0 (14.0, 45.0) | 0.0123^3^ |
| Gender (M/F) | 313 (59.6%)/212 (40.4%) | 294 (59.4%)/201 (40.6%) | 19 (63.3%)/11 (36.7%) | 0.6694^2^ |
| BMI (Kg/m^2^) | 23.4 (10.7, 35.8) | 23.4 (10.7, 35.8) | 24.4 (16.5, 29.4) | 0.2022^3^ |
| ICU stay (days) | 2.0 (0.0, 30.0) | 2.0 (0.0, 30.0) | 2.0 (1.0, 12.0) | 0.9179^4^ |
| Amylase (IU/L) | 80.0 (0.0, 1831.0) | 77.5 (0.0, 1831.0) | 140.0 (13.0, 958.0) | 0.0095^4^ |
| Lipase (IU/L) | 34.5 (4.0, 1002.0) | 36.0 (4.0, 1002.0) | 26.0 (10.0, 323.0) | 0.9264^4^ |
| P-PASS total | 16.0 (10.0, 23.0) | 16.0 (10.0, 23.0) | 16.0 (12.0, 19.0) | 0.8037^3^ |
| Preservation solution  - UW  - CS  - HTK  - IGL-1 | 271 (51.6%)  99 (18.9%)  8 (1.5%)  147 (28%) | 250 (50.5%)  93 (18.8%)  8 (1.6%)  144 (29.1%) | 21 (70.0%)  6 (20.0%)  -  3 (10.0%) | 0.0991^2^ |
| Pancreas CIT* (hours) | 10.0 (4.0, 20.0) | 9.5 (4.0, 20.0) | 11.8 (5.0, 17.0) | 0.0013^3^ |
| RECIPIENT |  |  |  |  |
| Age (years) | 41.0 (14.0, 62.0) | 41.0 (14.0, 62.0) | 41.0 (24.0, 53.0) | 0.9173^1^ |
| Gender (M/F) | 330 (62.9%)/195 (37.1%) | 311 (62.8%)/184 (37.2%) | 19 (63.3%)/11 (36.7%) | 0.9557^2^ |
| BMI (Kg/m^2^) | 23.0 (16.0, 37.5) | 23.0 (16.0, 37.5) | 24.9 (17.6, 33.2) | 0.0858^1^ |
| Type of DM  - DM 1  - DM 2  - Others | 516 (98.3%)  3 (0.6%)  6 (1.1%) | 486 (98.2%)  3 (0.6%)  6 (1.2%) | 30 (100.0%)  -  - | 0.7577^2^ |
| DM *vintage* (years) | 26.0 (6.5, 50.0) | 26.0 (6.5, 50.0) | 30.0 (14.0, 49.0) | 0.1215^3^ |
| Dialysis *vintage* (months) | 24.0 (0.0, 146.0) | 24.0 (0.0, 146.0) | 20.9 (1.7, 114.3) | 0.8047^3^ |
| Type of dialysis  - Predialysis  - Peritoneal  - Hemodialysis  - None | 63 (12%)  108 (20.6%)  291 (55.4)  63 (12%) | 62 (12.5%)  104 (21.0%)  273 (55.2%)  56 (11.3%) | 1 (3.3%)  4 (13.3%)  18 (60.0%)  7 (23.3%) | 0.0967^2^ |
| Transplant type  - SPK  - PAK  - PA  - Retransplant | 444 (84.6%)  30 (5.7%)  3 (0.6%)  48 (9.1%) | 424 (85.7%)  26 (5.3%)  3 (0.6%)  42 (8.5%) | 20 (66.7%)  4 (13.3%)  -  6 (20.0%) | 0.0332^2^ |

Donor factors: ^1^Fisher Exact p-value; ^2^Chi-Square p-value; ^3^Equal variance two sample t-test; ^4^Kruskal-Wallis p-value.

Recipient factors: ^1^Equal variance two sample t-test; ^2^Chi-Square p-value; ^3^Kruskal-Wallis p-value.

Continuous variables are expressed as median (range) and categorical variables as frequencies (percentages).

BMI, body mass index; cDCD, controlled donation after circulatory death; CS, Celsior; CIT, cold ischemia time; CVA, cerebrovascular accident; DBD, donation after brain death; DD, duodenoduodenostomy; DJ, duodenojejunostomy; DM, Diabetes Mellitus; F, female; HTK, Histidine-Tryptophan-Ketoglutarate; IGL-1, Institut Georges Lopez-1; ICU, intensive care unit; M, male; PPASS, preprocurement pancreas suitability score; PAK, Pancreas After Kidney; PA, Pancreas Transplant Alone; SPK, Simultaneous Pancreas-Kidney; UW, University of Wisconsin.

*CIT is the interval between the initiation of organ perfusion with cold preservation solution in the donor and the onset of reperfusion in the recipient.
