# Supplementary material for: Duodenoduodenostomy as an Attractive Option for Exocrine Drainage in Pancreas Transplantation: Insights From a Single-Center Cohort
Source: Transpl Int. 2025 Nov 3;38:15430. doi: 10.3389/ti.2025.15430 (PMC12620303; doi:10.3389/ti.2025.15430)
Supplement: Supplementary file 2 [file Table2.docx]

**Supplementary Table 2.** Graft failure factors

|  | Total  (n = 525) | Functional graft  (n = 389) | Failed graft  (n = 136) | *P* value |
| --- | --- | --- | --- | --- |
| DONOR |  |  |  |  |
| Type of donor  - DBD  - cDCD III | 493 (93.9%)  32 (6.1%) | 363 (93.3%)  26 (6.7%) | 130 (95.6%)  6 (4.4%) | 0.4098^1^ |
| Cause of death  - Trauma  - CVA  - Anoxic damage  - Euthanasia  - Others | 251 (47.8%)  207 (39.4%)  41 (7.8%)  4 (0.8%)  22 (4.2%) | 191 (49.1%)  149 (38.3%)  30 (7.7%)  3 (0.8%)  16 (4.1%) | 60 (44.1%)  58 (42.6%)  11 (8.1%)  1 (0.7%)  6 (4.4%) | 0.9026^2^ |
| Age (years) | 33.0 (9.0, 59.0) | 32.0 (9.0, 59.0) | 34.0 (13.0, 54.0) | 0.3022^3^ |
| Gender (M/F) | 313 (59.6%)/212 (40.4%) | 235 (60.4%)/154 (39.6%) | 78 (57.4%)/58 (42.6%) | 0.5315^2^ |
| BMI (Kg/m^2^) | 23.4 (10.7, 35.8) | 23.4 (10.7, 35.8) | 23.5 (16.3, 31.0) | 0.2714^3^ |
| ICU stay (days) | 2.0 (0.0, 30.0) | 2.0 (0.0, 30.0) | 2.0 (1.0, 20.0) | 0.8859^4^ |
| Amylase (IU/L) | 80.0 (0.0, 1831.0) | 79.0 (6.0, 1831.0) | 85.0 (0.0, 796.0) | 0.7414^4^ |
| Lipase (IU/L) | 34.5 (4.0, 1002.0) | 36.0 (5.0, 451.0) | 33.0 (4.0, 1002.0) | 0.8221^4^ |
| P-PASS total | 16.0 (10.0, 23.0) | 16.0 (10.0, 23.0) | 16.0 (10.0, 21.0) | 0.2482^3^ |
| Preservation solution  - UW  - CS  - HTK  - IGL-1 | 271 (51.6%)  99 (18.9%)  8 (1.5%)  147 (28%) | 191 (49.1%)  69 (17.7%)  3 (0.8%)  126 (32.4%) | 80 (58.8%)  30 (22.1%)  5 (3.7%)  21 (15.4%) | 0.0003^2^ |
| Pancreas CIT* (hours) | 10.0 (4.0, 20.0) | 9.0 (4.0, 18.5) | 10.3 (4.0, 20.0) | 0.0036^3^ |
| RECIPIENT |  |  |  |  |
| Age (years) | 41.0 (14.0, 62.0) | 41.0 (14.0, 62.0) | 40.5 (15.0, 57.0) | 0.0365^1^ |
| Gender (M/F) | 330 (62.9%)/195 (37.1%) | 250 (64.3%)/139 (35.7%) | 80 (58.8%)/56 (41.2%) | 0.2581^2^ |
| BMI (Kg/m^2^) | 23.0 (16.0, 37.5) | 22.7 (16.0, 34.0) | 23.9 (17.6, 37.5) | 0.0275^1^ |
| Type of DM  - DM 1  - DM 2  - Others | 516 (98.3%)  3 (0.6%)  6 (1.1%) | 382 (98.2%)  3 (0.8%)  4 (1.0%) | 134 (98.5%)  -  2 (1.5%) | 0.5427^2^ |
| DM *vintage* (years) | 26.0 (6.5, 50.0) | 27.0 (10.0, 50.0) | 25.5 (6.5, 44.0) | 0.1368^3^ |
| Dialysis *vintage* (months) | 24.0 (0.0, 146.0) | 24.0 (0.0, 146.0) | 24.2 (1.0, 114.3) | 0.8709^3^ |
| Type of dialysis  - Predialysis  - Peritoneal  - Hemodialysis  - None | 63 (12%)  108 (20.6%)  291 (55.4)  63 (12%) | 48 (12.3%)  92 (23.7%)  211 (54.2%)  38 (9.8%) | 15 (11.0%)  16 (11.8%)  80 (58.8%)  25 (18.4%) | 0.0034^2^ |
| Transplant type  - SPK  - PAK  - PA  - Retransplant | 444 (84.6%)  30 (5.7%)  3 (0.6%)  48 (9.1%) | 338 (86.9%)  20 (5.1%)  1 (0.3%)  30 (7.7%) | 106 (77.9%)  10 (7.4%)  2 (1.5%)  18 (13.2%) | 0.0509^2^ |

Donor factors: ^1^Fisher Exact p-value; ^2^Chi-Square p-value; ^3^Equal variance two sample t-test; ^4^Kruskal-Wallis p-value.

Recipient factors: ^1^Equal variance two sample t-test; ^2^Chi-Square p-value; ^3^Kruskal-Wallis p-value.

Continuous variables are expressed as median (range) and categorical variables as frequencies (percentages).

BMI, body mass index; cDCD, controlled donation after circulatory death; CS, Celsior; CIT, cold ischemia time; CVA, cerebrovascular accident; DBD, donation after brain death; DD, duodenoduodenostomy; DJ, duodenojejunostomy; DM, Diabetes Mellitus; F, female; HTK, Histidine-Tryptophan-Ketoglutarate; IGL-1, Institut Georges Lopez-1; ICU, intensive care unit; M, male; PPASS, preprocurement pancreas suitability score; PAK, Pancreas After Kidney; PA, Pancreas Transplant Alone; SPK, Simultaneous Pancreas-Kidney; UW, University of Wisconsin.

*CIT is the interval between the initiation of organ perfusion with cold preservation solution in the donor and the onset of reperfusion in the recipient.
